# FRET-Mediated Long-Range Wavelength Transformation by Photoconvertible Fluorescent Proteins as an Efficient Mechanism to Generate Orange-Red Light in Symbiotic Deep Water Corals

**DOI:** 10.3390/ijms18071174

**Published:** 2017-07-04

**Authors:** Elena Bollati, Daniel Plimmer, Cecilia D’Angelo, Jörg Wiedenmann

**Affiliations:** 1 Coral Reef Laboratory, University of Southampton, Waterfront Campus, European Way, Southampton SO143ZH, UK; e.bollati@soton.ac.uk (E.B.); dp5g11@southamptonalumni.ac.uk (D.P.); C.D’angelo@soton.ac.uk (C.D.); 2 Institute for Life Sciences (IFLS), University of Southampton, Highfield Campus, Southampton SO171BJ, UK

**Keywords:** fluorescent protein, fluorescence, photoconversion, coral, mesophotic, FRET

## Abstract

Photoconvertible fluorescent proteins (pcRFPs) are a group of fluorophores that undergo an irreversible green-to-red shift in emission colour upon irradiation with near-ultraviolet (near-UV) light. Despite their wide application in biotechnology, the high-level expression of pcRFPs in mesophotic and depth-generalist coral species currently lacks a biological explanation. Additionally, reduced penetration of near-UV wavelengths in water poses the question whether light-driven photoconversion is relevant in the mesophotic zone, or whether a different mechanism is involved in the post-translational pigment modification in vivo. Here, we show in a long-term mesocosm experiment that photoconversion in vivo is entirely dependent on near-UV wavelengths. However, a near-UV intensity equivalent to the mesophotic underwater light field at 80 m depth is sufficient to drive the process in vitro, suggesting that photoconversion can occur near the lower distribution limits of these corals. Furthermore, live coral colonies showed evidence of efficient Förster Resonance Energy Transfer (FRET). Our simulated mesophotic light field maintained the pcRFP pool in a partially photoconverted state in vivo, maximising intra-tetrameric FRET and creating a long-range wavelength conversion system with higher quantum yield than other native RFPs. We hypothesise that efficient conversion of blue wavelengths, abundant at depth, into orange-red light could constitute an adaptation of corals to life in light-limited environments.

## 1. Introduction

Reef corals owe a large proportion of their striking green, red, and purple-blue colouration to green fluorescent protein (GFP)-like host pigments (FPs) found in the coral host [1,2,3,4]. The pigment concentrations of up to 7% of the total soluble protein content in coral tissue [2] are achieved by high-level expression of the encoding multicopy genes [5]. The first representative of this protein family, GFP, was described from the jellyfish *Aequorea victoria* [6] and more were subsequently isolated from other Anthozoa [7,8,9,10] and Scleractinia [2,3]. The GFP structure consists of an 11-stranded β-can [11] surrounding a fluorescent chromophore [12], and is widely conserved across a variety of taxa including reef corals [13,14]. Structural studies revealed that the majority of Anthozoan FPs, unlike the monomeric GFP [11], is assembled in tetramers [15].

The tripeptide chromophore is synthesised autocatalytically [16] in a process that requires molecular oxygen but no enzymes or cofactors [17]. The colour diversity of FPs can be attributed to modifications of the GFP-type chromophore and altered interactions with the surrounding protein scaffold [15,18]. Notably, an additional oxidation step modifying the GFP-type chromophore results in the emergence of red fluorescence, as observed in DsRed and eqFP611 [7,9]. In photoconvertible red fluorescent reef coral pigments such as Kaede or EosFP, the post-translational modification of the GFP-type chromophore is driven by exposure to near-ultraviolet (near-UV) light around 390 nm [14,19,20,21]. Protein pigments characterised by this photoconversion process can be found in a number of Anthozoa species [2,19,20,22] and have been termed “photoconvertible red fluorescent proteins” (pcRFPs). The photoconversion mechanism is associated with cleavage of the peptide backbone between the Cα and Nα of His62 [14,21], the first residue of a His-Tyr-Gly tripeptide that forms the chromophore of all so far known pcRFPs [2]. Although this mechanism has found wide biotechnological application [19,20,23,24,25], the biological function and ecological relevance of photoconversion in this pigment group remains unexplained.

A photoprotective role has been demonstrated for certain FPs and biochemically related non-fluorescent chromoproteins in sea anemones and corals living in symbiosis with dinoflagellate algae [5,26,27,28]. The predominant localisation of FPs and homologous non-fluorescent chromoproteins in the ectoderm above the symbiont-harbouring endoderm [28,29] supports the experimental observations. Furthermore, D’Angelo and co-workers [30] found that many cyan, green and DsRed-type red FPs are regulated at the transcriptional level by the intensity of incident light, particularly in the blue spectral range [5,28,30]. High tissue concentrations of FPs are also found, however, in azooxanthellate Anthozoa in a range of habitats not prone to light stress, including the deep sea [10,31], and in zooxanthellate mesophotic corals [2,32,33,34]. In the latter group, FPs are constitutively expressed independently of the level of light exposure [33,35,36] and have been suggested to facilitate photosynthesis of symbiotic algae in low light conditions [26,27]. pcRFPs are commonly found in corals of the Faviina suborder with a preference for lower light habitats [37], they have been reported from mesophotic depths [33] and their regulation is independent of light [35]. Furthermore, pcRFPs exhibit efficient non-radiative energy transfer via FRET between green and red chromophores in subunits of partially converted tetramers [20]. This is promoted by the strong overlap of the emission spectrum of the green precursor chromophore with the absorption/excitation spectrum of the photoconverted red form, and by the close proximity between the chromophores within the tetrameric assembly [14,19,20,38]. Therefore, the formation of the chromophore and the photophysical properties of pcRFPs are distinct from those of red-emitting FPs from shallow water corals.

Green-to-red photoconversion might thus represent an important part of the adaption of mesophotic corals to life in low light environments. However, the blue-green wavelengths that dominate these light fields fall mostly beyond the photoconversion action spectrum of pcRFPs measured in vitro using recombinantly produced proteins [20]. Hence, the question arises whether photoconversion of coral pigments at greater depth is at all of ecological relevance. To investigate whether the unique photophysical properties of pcRFPs represent an adaptation to life in low light environments, we explored the effects of a simulated deep-water light field on corals containing pcRFPs. Specifically, we assessed whether the near-UV light levels prevailing within the depth range of such corals are sufficient to induce green-to-red photoconversion. Finally, we investigated the possibility of FRET as a mechanism to fine-tune the internal light environment of the colonies. Our findings help place the photoconversion mechanism in an ecological context, a prerequisite to understanding the function of pcRFPs in the coral holobiont.

## 2. Results

### 2.1. Effects of Light Quality on Photoconvertible Red Fluorescent Proteins (pcRFPs)

#### 2.1.1. Changes in Live Tissue Fluorescence over Time

In order to investigate the spectral dependence of photoconversion in live corals, colonies of *Montastraea cavernosa* and *Echinophyllia* sp. were exposed to 412 nm, 448 nm and 476 nm light from light-emitting diodes (LEDs) for >120 days. Fluorescence emission and excitation from the colony coenosteum were monitored in vivo throughout the exposure period.

The intensity of the LEDs used for the light treatment was adjusted to levels at which the amount of photons efficient for photoconversion fell within the range that corals experience over a depth gradient in their natural habitat. Specifically, the 412 nm LED set to 60 μmol photons·m^−2^·s^−1^ has a photoconversion potential equivalent to that of our Red Sea irradiance data at ~26 m, while the 448 nm LED was equivalent to ~81 m; the 476 nm LED had no photoconversion potential. For comparison, the photoconversion potential of 200 μmol photons·m^−2^·s^−1^ of metal halide was equivalent to ~37 m in the Red Sea.

**Figure 1:**
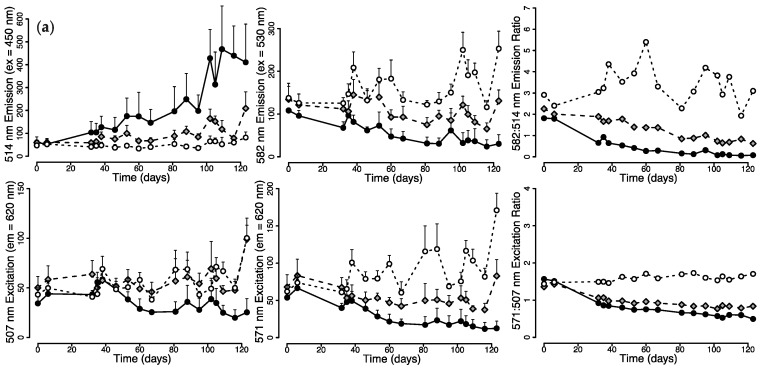

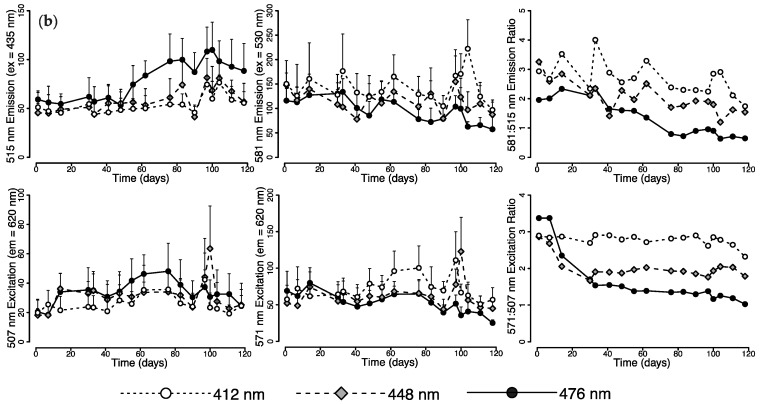
In vivo fluorescence of *M. cavernosa* and *Echinophyllia* sp. during 123 days exposure to 476, 448 and 412 nm light. (**a**) *M. cavernosa*: green emission (514 nm) measured with 450 nm excitation, red emission (582 nm) with 530 nm excitation, and 507 and 571 nm excitation measured for 620 nm emission; (**b**) *Echinophyllia* sp.: green emission (515 nm) measured with 435 nm excitation, red emission (581 nm) measured with 530 nm excitation, and 507 and 571 nm excitation measured for 620 nm emission. Mean + Standard Deviation (S.D.), *n* = 6.

Under 476 nm light illumination, green (514 nm) fluorescence emission of *M. cavernosa* increased with time, reaching ~6× pre-treatment values; this was accompanied by a decrease in red (582 nm) emission down to ~30% of pre-treatment fluorescence (Figure 1a). Values in the excitation maximum (571 nm) of red fluorescence also decreased over time to ~25% of initial values. In contrast, when red fluorescence emission was recorded, the excitation value at 507 nm corresponding to the excitation maximum of the green fluorescent chromophore showed only a slightly decreasing trend. *M. cavernosa* exposed to 448 nm light showed a smaller increase in green emission, while red emission, 507 nm red excitation and 571 nm red excitation showed only minor changes. Under 412 nm light illumination, green emission did not show pronounced changes, while an increase in red emission, 507 nm red excitation and 571 nm red excitation was observed (Figure 1a).

Similar trends were observed for *Echinophyllia*, with green fluorescence emission (515 nm, ex = 450 nm) increasing under 476 nm light. Red fluorescence emission (581 nm, ex = 530 nm) and excitation (571 nm, em = 620 nm) decreased down to ~50% of the initial values. In contrast, red fluorescence appeared essentially unchanged in comparison to pre-treatment values when excited in the green excitation band (507 nm). Colonies of *Echinophyllia* kept under 448 nm and 412 nm light, however, showed no major difference in the measured tissue fluorescence values during the experiment (Figure 1b).

The ratio of red-to-green fluorescence emission values provides an indication of the relative proportions of unconverted (green) and converted (red) protein. For both *M. cavernosa* (Figure 1a) and *Echinophyllia* (Figure 1b), the ratio remained relatively stable over time when the corals were exposed to 412 nm light, with values comparable to those of colonies kept under metal halide, while it showed a decreasing trend under 448 nm light and an even larger decrease under 476 nm light. The ratio between the red fluorescence excitation values recorded at 571 and 506 nm was also constant under 412 nm light, while it stabilised at a lower value under 448 and 476 nm light (Figure 1a,b).

#### 2.1.2. Microscopic and Spectroscopic Characterization of Tissue Fluorescence

**Figure 2:**
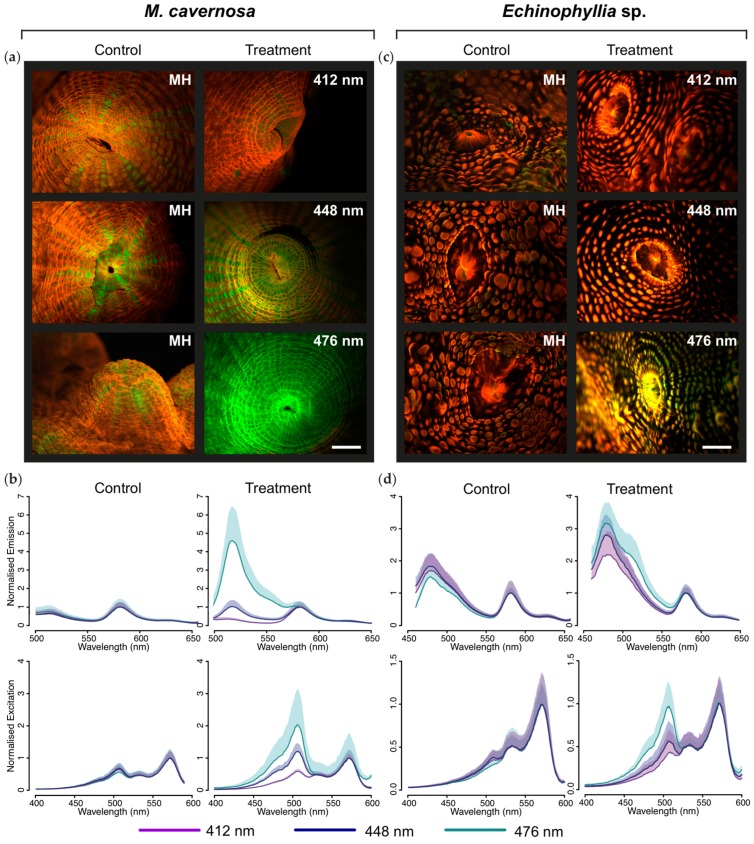
Fluorescence of *M. cavernosa* and *Echinophyllia* under metal halide or narrow-waveband light-emitting diode (LED): (**a**,**c**) Fluorescent micrographs of *M. cavernosa* (**a**), and *Echinophyllia* sp. (**c**) under metal halide (MH; control) or narrow-waveband LED (412 nm, 448 nm, 476 nm; treatment); scale bar = 2 mm; (**b**,**d**) Emission spectra obtained with: 450 nm excitation for *M. cavernosa* (**b**), and 435 nm for *Echinophyllia* sp. (**d**) Excitation spectra obtained for 620 nm emission. Mean + S.D., *n* = 6. Spectra are normalised to intensity of the 582 nm peak.

Microscopic imaging of the control colonies of *M. cavernosa* kept under white metal halide light revealed prevalent red fluorescence across the coenosteum, with green fluorescence of the tentacles [2] contributing to the fluorescence of the polyps (Figure 2a). Spectroscopic measurements showed a red emission (excitation) peak at 582 (571) nm, along with a smaller green peak at 514 (507) nm (Figure 2b). *M. cavernosa* kept under 412 nm light had retained red coenosteum fluorescence, while green fluorescence was still visible exclusively in the oral region of polyps (Figure 2a); emission and excitation spectra matched those measured in the white light control colonies (Figure 2b). When kept under 476 nm LED, on the other hand, the coenosteum showed dominant green fluorescence (Figure 2a) and a corresponding increase in the green emission and excitation peak (Figure 2b). The colony exposed to 448 nm light appeared as an intermediate stage, with both red and green fluorescence observable across the tissue (Figure 2a); the emission and excitation spectrum were characterised by double peaks in the green and red regions (Figure 2b).

Prevalence of red fluorescence was also observed for colonies of *Echinophyllia* kept under metal halide or 412 nm LED (Figure 2c). The red emission (excitation) spectrum peaked at 581 (571) nm, and a small green excitation peak was detected at 507 nm. However, the emission spectrum showed a strong peak around 480 nm, indicating the contribution of cyan fluorescent pigments (Figure 2d). Kept under the 476 nm LED, the coral tissue appeared yellow in fluorescence micrographs, indicating a mixture of red and green fluorescence (Figure 2c). The emission spectrum showed an increase in the cyan peak relative to red emission values, as well as in the contribution of green fluorescence with a maximum around 507 nm visible as a pronounced shoulder on the 480 nm emission peak. The green excitation maximum was equal in magnitude to the red peak (Figure 2d). *Echinophyllia* sp. kept under 448 nm light showed an intermediate appearance between those under 476 and 412 nm.

#### 2.1.3. Purified pcRFPs

**Figure 3:**
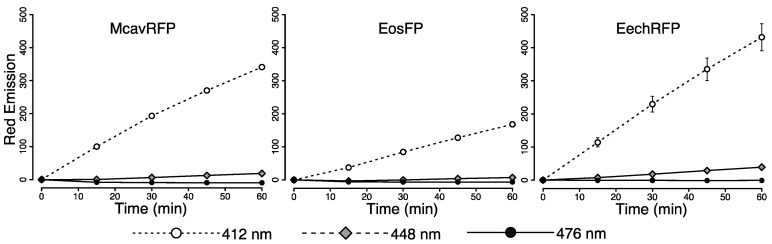
In vitro photoconversion of purified pcRFPs under 412, 448 and 476 nm light. Photoconversion measured as red (580–581 nm) fluorescence emission (ex = 530) every 15 min for 60 min under 60 μmol photons·m^−2^·s^−1^. Mean ± S.D., *n* = 3.

To test whether the changes in fluorescence of live coral tissue can be reproduced with purified pcRFPs in vitro, we produced the major pigments of *M. cavernosa* (McavRFP), *L. hemprichii* (EosFP) and *Echinophyllia echinata* (EechRFP) in a bacterial expression system. Aliquots of the purified proteins in their green, unconverted state were exposed to light from the same 412 nm, 448 nm and 476 nm LEDs. Photon flux was set to 60 μmol photons·m^−2^·s^−1^ to match the photon flux used in the in vivo experiment and the corresponding equivalent depths. Consistently with what observed in live colonies, 476 nm light was ineffective for photoconversion and no changes in red fluorescence emission (581–582 nm, ex = 530 nm) were observed after one hour. Exposure to 412 nm light was most efficient in producing the red fluorescent photoconversion product. In contrast, only a small increase in red emission was observed for pcRFPs under the 448 nm LED (Figure 3).

### 2.2. Photoconversion Along a Simulated Depth-Irradiance Gradient

**Figure 4:**
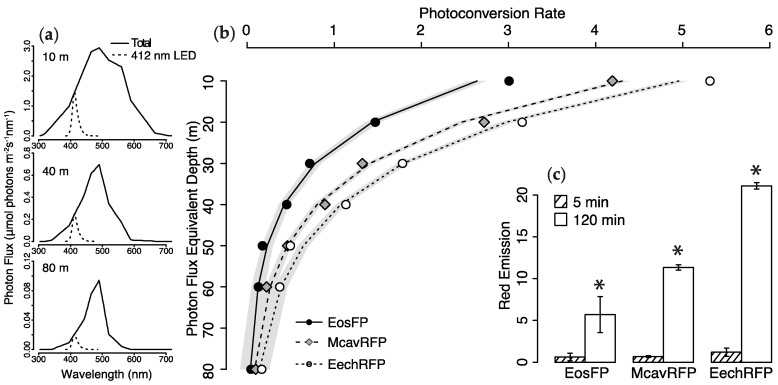
Photoconversion of purified McavRFP, EosFP and EechRFP along a simulated depth gradient. (**a**) Scaling of 412 nm LED to photon flux measurements at 10, 40 and 80 m in the Red Sea [33]; values for total photon flux and scaled 412 nm photon flux are provided in Table S1a; (**b**) Photoconversion rates of pcRFPs along 412 nm photon flux gradient. Points represent the slope of the linear regression equation obtained measuring red fluorescence emission (580–581 nm, ex = 530 nm) during photoconversion every 1 min for 2 h (*n* = 3 conversions per protein per photon flux). Lines represent fitted exponential decay function ±95% c.i. Details and results of statistical analysis are provided in Table S1b; (**c**) Red fluorescence emission (580–581 nm, ex = 530 nm) of purified pcRFPs after 2 h exposure to 0.5 μmol photons·m^−2^·s^−1^ from 412 nm LED, comparable to the photon flux in the same range at 80 m depth in the Red Sea. Mean ± S.D., *n* = 3. Stars indicate *p* < 0.001 in post-hoc comparison (Tukey’s Honest Significant Difference) following Repeated Measures Analysis of Variance (RM-ANOVA), F_(5,8)_ = 2364.29, *p* < 0.001.

To quantify the photoconversion potential of light within the depth range of mesophotic corals, we exposed aliquots of purified, unconverted McavRFP, EosFP, and EechRFP to a series 412 nm light treatments with intensities comparable to those measured along a depth gradient in the Red Sea (Figure 4a). At all intensities, the abundance of the red chromophore measured as fluorescence emission intensity at 580–581 nm (ex = 530 nm) was found to increase linearly with time (Table S1a). For all proteins, photoconversion was fastest at intensities corresponding to the 10 m depth light field and decreased with decreasing intensity. At all intensities, photoconversion was fastest for EechRFP, followed by McavRFP and EosFP.

Since the increase in red emission was essentially linear over the duration of the experiment, the slope of fitted regression lines represents the photoconversion rate for each protein at the specified photon flux. When plotted against the simulated depth gradient, the decrease in photoconversion rates can be described by an exponential function (Figure 4b). The model equations and results of statistical analysis are given in Table S1b.

Red fluorescence of the purified protein solutions was compared before and after a 2 h exposure to 0.5 μmol photons·m^−2^·s^−1^ in the 412 nm band, a photon flux corresponding to a simulated depth of 80 m. The change in red emission over the exposure period was found to be statistically significant (RM-ANOVA, F_(5,8)_ = 2364.29, *p* < 0.001), and post-hoc comparison with Tukey’s Honest Significant Difference (HSD) test showed that this was the case for all three pcRFPs under study (Figure 4c).

### 2.3. Wavelength Transfer by Förster Resonance Energy Transfer (FRET)

**Figure 5:**
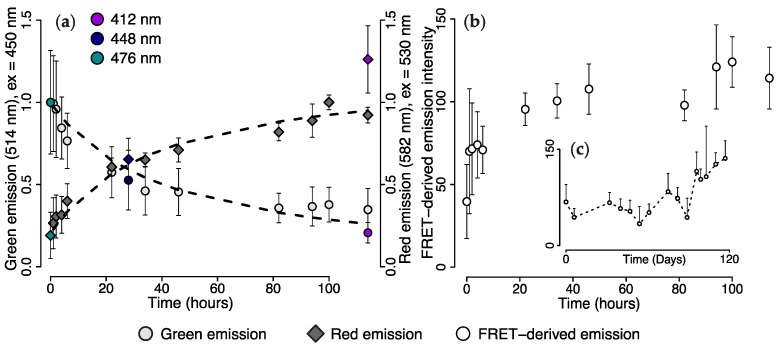
In vivo photoconversion of green *M. cavernosa* under 412 nm light. The colony had been previously kept under 476 nm light for >123 days. (**a**) Green (514 nm) emission measured over 110 h with 450 nm excitation light; red (582 nm) emission measured with 530 nm excitation light. Dashed lines show fitted second order reaction rate models; model equations and results of statistical analysis are provided in Table S2. Coloured data points show values after long-term exposure to 412, 448 and 476 nm light; (**b**,**c**) FRET-derived emission intensity (582 nm) measured with 450 nm excitation light and corrected according to Equations (2)–(4). Values calculated for short-term exposure to 412 nm LED after acclimation with 476 nm LED, means ± S.D., *n* = 5 (**b**); and for long-term exposure to 412 nm LED after acclimation with white metal halide, means + S.D., *n* = 6 (**c**).

To investigate whether FRET between the green and the red fluorescent chromophores occurred in live colonies, we placed *M. cavernosa* from the 476 nm light treatment under the 412 nm LED and monitored tissue fluorescence over 10 days. Green (514 nm) emission (ex = 450 nm) decreased within 34 h to 50% of the initial values before reaching a plateau. Red (582 nm) emission (ex = 530 nm) showed the opposite trend, with a value up to five-fold higher being reached after 100 h (Figure 5a). These values were normalised for use as proxy for red and green chromophore concentration, which appeared consistent with the kinetics of a second order reaction with a rate coefficient of 0.03 (non-linear least squares, *p* < 0.001, Figure 5A, Table S2).

To estimate FRET, we used the FRET-sensitised emission measurement—i.e., emission of the red chromophore (582 nm) upon blue light excitation (450 nm)—to calculate the FRET-derived emission intensity (Figure 5b). This measurement indicated that FRET contribution increased very rapidly during the first hour of the experiment. The rate of increase started to reduce after ~22 h; the maximal FRET contribution values were reached after 100 h (Figure 5b). The same calculation was applied to the data obtained from the long-term 412 nm light treatment, which showed that FRET-derived emission was stable at a low level for the first half of the experiment, and increased in the second half up to maximum values comparable to those reached in the short-term experiment (Figure 5c).

### 2.4. Efficiency of FRET-Mediated Wavelength Transfer

**Figure 6:**
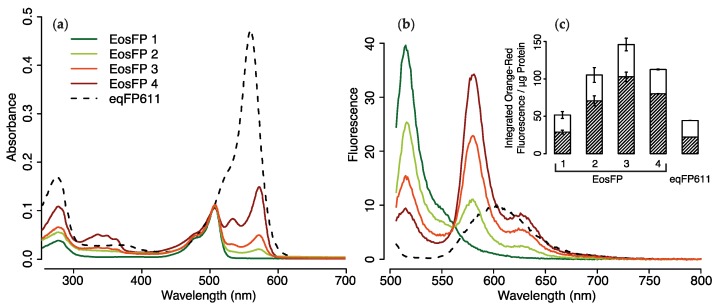
Spectra of purified EosFP at various photoconversion stages (1 = green EosFP; 2,3,4 = partially converted EosFP) vs. eqFP611. Absorbance (**a**); and fluorescence (**b**) spectra; and integrated fluorescence values (**c**) of protein aliquots diluted in PBS pH 7.4 to 0.1 absorbance at 506 nm. Fluorescence spectra obtained with excitation set to 506 nm. Integrated fluorescence normalised to μg functional protein, concentrations are provided in Table S3. Integrated orange–red fluorescence values measured between 550 and 700 nm, shaded areas represent 560–610 nm portion; (**a**,**b**) Mean, *n* = 3; (**c**) mean ± S.D., *n* = 3.

To assess the capacity of pcRFPs to convert blue-green light into yellow-red fluorescence in vitro, we compared the wavelength transfer efficiency of EosFP at various photoconversion stages with those of the red fluorescent protein eqFP611. Upon excitation at 506 nm, partially converted EosFP samples had considerably higher emission intensity in the 550–700 and 560–610 nm range per μg of functional protein as compared to eqFP611 (Figure 6a–c). Partially photoconverted EosFP with a >3:1 ratio of green to red chromophores (EosFP 2) generated orange-red emission that was ~2.4 times higher than the same protein amount of eqFP611. At a ~1:1 ratio of green and red chromophores of EosFP (EosFP 3), the wavelength conversion of EosFP per μg of protein was ~3.3-fold higher than that of eqFP611 (Figure 6c, Table S3).

## 3. Discussion

With growing anthropogenic pressure on shallow water reefs [39], mesophotic ecosystems have been suggested as potential refugia for coral communities [40,41]. Since the light environment changes dramatically with depth, the knowledge of how corals and their symbiotic algae can cope with low irradiances and narrow spectra is crucial to understand the capability of various species to survive at greater depths.

pcRFPs have been mostly reported from depth generalist corals [3] and were encountered in individuals collected from mesophotic depths [33]. Their expression is not regulated at the transcriptional level by light intensity as compared to counterparts in shallow water corals [5,30,35]. Together with the finding that their red fluorescence was under positive selection during the evolution of coral FPs [42], these observations suggest that these pigments could be part of an adaptation strategy to life in low light environments [27,43]. The red fluorescent chromophore of pcRFPs, integral to their function, is generated by a post-translational modification of a green fluorescent precursor in a photoconversion process induced by near-UV light. However, while blue-green light penetrates down to the lower limits of the euphotic zone, near-UV wavelengths required for photoconversion and maintenance of red fluorescence in coral colonies are strongly attenuated by the water column, dropping to very low levels at mesophotic depths [43,44,45]. Therefore, it is questionable whether a light-driven photoconversion is at all relevant for the adaptation of coral to their natural light environment.

### 3.1. Effects of Spectral Quality on Photoconversion of pcRFPs

Using narrow-waveband LED technology in an experimental study, we were able to address this question and assess the effects of different parts of the light spectrum on the photoconversion of coral pcRFPs, both in vivo and in vitro. Our >120 days mesocosm experiment showed that the spectral quality has a strong influence on fluorescence of pcRFP-containing corals. Under the narrow-band LED spectrum, only corals exposed to 412 nm light were able to retain ratios of red-to-green fluorescence comparable to those observed under white light; corals exposed to 448 and 476 nm light showed increasing levels of green fluorescence, indicating accumulation of unconverted pcRFPs in the tissue.

The 476 nm light used in our experiment had no spectral overlap with the photoconversion action spectrum [19,20], thus being ineffective in inducing the post-translational modification both in vivo and in vitro. The observed increase in green fluorescence reflects the accumulation of unconverted protein in the tissue, caused by failure of newly synthesised protein to photoconvert into the red form. At the same time, red fluorescence decreased due to progressive decay of the converted protein fraction, previously shown to occur in the dark with a half-life of ~20 days [35], as well as photobleaching caused by prolonged excitation of the chromophore with 476 nm light treatment [35,46]. Exposing purified proteins to the same light conditions yielded comparable results to those obtained for live colonies.

We exposed the purified pcRFPs to a series of different intensities of 412 nm light to simulate the attenuation of downwelling near-UV irradiance in coral reef habitats [47]. While differences in the individual photoconversion rates exist among the pcRFPs under study, all of them showed an exponential decrease, thus appearing to depend entirely on photon flux in the near UV range. As our mesocosm experiment results show that photoconversion is near-UV dependent in vivo, we assume this exponential trend to also be relevant for live corals in situ. Importantly, even at a photon flux as low as 0.5 μmol photons·m^−2^·s^−1^, corresponding to values measured at 80 m in the Red Sea [33], we observed successful albeit slow photoconversion of EosFP, McavRFP, and EechRFP after just 2 h of exposure. This suggests that despite stronger attenuation of UV and near-UV compared to blue-green wavelengths by the water column, photoconversion of pcRFPs can indeed happen in the mesophotic zone.

### 3.2. Wavelength Transformation via FRET

As demonstrated by our in vitro experiments, partially converted pcRFP tetramers represent ideal donor-acceptor FRET pairs due to the close proximity of green and red chromophores in the tetrameric assemblage of the protein [14,20]. As a result, green emission of the individual tetramer disappears in presence of even a single photoconverted subunit in EosFP [20]. Consequently, the photoconversion process will alter the emission properties of pcRFPs, starting with a green light emitting pigment that absorbs blue-green light, and then shifting to a mixture of green and red emitting chromophores that still absorb mostly blue-green light but emit predominantly orange-red light. This appears as an intriguing mechanism to transform parts of the blue-green wavelengths that dominate the underwater light field at greater depths into orange-red wavelengths. In this way, the corals may compensate for the depth-dependent attenuation of longer wavelength light and produce an internal light field that more closely resembles the conditions in shallow water.

To assess whether FRET could be a mechanism for wavelength transformation in live *M. cavernosa*, we calculated the FRET-derived emission for a colony pre-treated with 476 nm light undergoing photoconversion. This was achieved by measuring the emission of the red acceptor upon excitation of the green donor (FRET-sensitised emission) and applying a correction to remove spectral contribution by donor emission and by direct acceptor excitation, an approach analogous to the three-filter methodology used in FRET microscopy [48,49,50].

With progressive appearance of red fluorescence during photoconversion we observed a rapid increase in FRET-derived emission, which can be attributed to the increasing acceptor concentration [38]. In live colonies, a stabilisation of FRET levels can be expected from the steady-state equilibrium that results from the continuous depletion of red acceptor chromophores by photobleaching and protein turnover [20,35] and the constitutive expression of pcRFPs, which supplements the pool of green donor chromophores [35]. The maximum FRET value for a colony in a given light field will thus depend on: (i) the number of photons available to excite the red and the green chromophores, respectively, causing photobleaching directly and via FRET; (ii) light intensity in the spectral range efficient for photoconversion; and (iii) the protein turnover rate.

After 22 h under a photon flux of 60 μmol·m^−2^·s^−1^ provided by the 412 nm LED, FRET-derived emission began to saturate and it reached its maximum after 100 h. These values were comparable with those obtained under the same light condition in the long-term exposure experiment. While the ratio of green and red fluorescent chromophores in the individuals from the 412 nm treatment is comparable with the ratio determined for the corals cultured under metal halide illumination, both green and red fluorescence emission is lower in the latter corals. This is likely due to the broad spectrum of the metal halide light, which excites both chromophore types very well, resulting in increased levels of photobleaching [20] and a steady-state equilibrium that contains fewer functional chromophores.

Therefore, when colonies acclimated to the metal halide spectrum were transferred to the LED experiments, the steady-state equilibrium of green and red chromophores characteristic of the 412 nm light condition only became evident after complete turnover of the pool of pigments present at the beginning of the experiment, a slow process that occurs with a half-life of about three weeks [35].

With increasing simulated depth, both photoconversion and photobleaching rates are reduced, producing colonies with a mixture of green and red chromophores. Our in vitro experiments with EosFP show that most efficient conversion of blue-green excitation wavelengths into orange-red fluorescence emission is achieved at roughly equal concentrations of green donor and red acceptor chromophores. Although a 1:3 red:green configuration within a single EosFP tetramer is sufficient to produce efficient FRET [20], in more complex systems, such as purified proteins in solution or native proteins in animal tissue, a 1:1 ratio maximises the number of partially converted tetramers, thereby achieving the highest potential for FRET-mediated wavelength conversion. Such a ratio resulted from the long-term exposure of *M. cavernosa* to the 448 nm LED. The photoconversion capacity of this light source at a photon flux of 60 μmol·m^−2^·s^−1^ is comparable to that of the Red Sea light field at ~80 m depth. Hence, the results of this experiment provide evidence that FRET-derived production of orange-red light is promoted in the mesophotic depth range, despite the reduced amount of light effective for photoconversion.

In pcRFPs, FRET-mediated fluorescence with excitation/emission maxima of 506/581 nm results in a Stokes shift of 75 nm and, hence, efficient short-to-long wavelength transfer. This Stokes shift is up to three times larger compared to red fluorescent proteins from shallow water corals [3,5], suggesting that FRET-mediated wavelength transformation is an efficient mechanism to supplement the corals and their symbiotic algae with orange-red light that becomes increasingly rare at greater depth. To assess this hypothesis, we compared the potential for blue-green to yellow-red wavelength conversion of partially photoconverted EosFP with that of the non-photoconvertible red protein eqFP611 [9]. This protein was selected for its absorption maximum similar to that of red EosFP, the high degree of red fluorophore maturation, the bright fluorescence and the exceptionally large Stokes shift of 52 nm [9]. We found that a partially converted EosFP with a ~1:1 ratio of red:green chromophores is over 3× more efficient at converting blue-green light into the 550–700 nm range (>6× in the 560–610 nm range) than the same amount of eqFP611. Considering the metabolic efforts required to reach the high pigment concentrations found in coral tissue [2,5], our results indicate that FRET-mediated wavelength transformation by partially converted pcRFPs is indeed an energetically favourable mechanism to produce orange-red light.

Remarkably, efficient FRET coupling within the tetrameric assemblage has also been found in the red fluorescent protein drFP583 (DsRed) from the disc anemone *Discosoma* sp. [7,51]. In this protein, a mixture of green and red chromophores is generated during the maturation process of the red chromophore, driven by a light-independent oxidation of the chromophore precursor. While the fluorescence properties of both the green and the red chromophores of DsRed and pcRFPs are similar, they have evolved independently from a green fluorescent common ancestor [3]. Notably, *Symbiodinium*-associated disc anemones such as *Discosoma* sp. often dwell in shaded habitats [52] which suggest that FRET-mediated wavelength transformation has evolved as an adaptation to light-limited habitats at least twice.

It has been suggested that fluorescent pigments in mesophotic corals such as *Leptoseris* sp. act to modify the quality of light available to the symbionts [27,33,42]. Our study demonstrates a remarkable mechanism by which pcRFPs can fine-tune the internal light climate of symbiotic corals along the steep gradient of light quantity and quality that characterises their habitat. Further work should improve our understanding of the significance of orange-red light generated by the FRET-mediated wavelength transfer for the adaptation to low-light environments.

## 4. Materials and Methods

### 4.1. Sample Culture and Aquarium Set up

Colonies of *M. cavernosa* (Linnaeus, 1767) and *Echinophyllia* sp. Klunzinger 1879 were cultured and propagated by fragmentation in the Coral Reef Laboratory mesocosm facility [36]. Prior to the start of the experiment, fragments were kept under a 400 W metal halide lamp (AquaMedic UK, Coalville, UK). To test the influence of the spectral quality of incident light on the photoconversion process, the corals were placed under Aquaray LED strips: (1) Aquaray NUV (emission peak wavelength: 412 nm); (2) Aquaray Fiji Blue (emission peak wavelength: 448 nm); and (3) Aquaray Reef Blue (emission peak wavelength: 476 nm) (TMC, London, UK). LED emission spectra were measured with a USB4000 modular spectrophotometer (Ocean Optics, Dunedin, FL, USA) and are provided in Figure S1. PVC pipes were attached under the individual LEDs to restrict the exposure of the replicate corals to stray light from neighbouring LEDs to negligible levels. Photon flux was set to ~50–60 μmol photons·m^−2^·s^−1^ as measured with a PAR light meter at water surface level (LI-COR, Lincoln, NE, USA). Specimens were acclimatised to the narrow-waveband light regime through progressive increase in daily exposure time over 11 days (1 to 4 h over 3 days, followed by 4 days at 4 h, then 2 h increase daily), after which the photoperiod was set to 12 h light:12 h dark. Colonies were kept under experimental conditions for a total of 123 days including acclimation time.

### 4.2. Live Colony Fluorescence

In vivo measurements of fluorescence emission/excitation were performed using a fluorescence spectrophotometer (Cary, Varian, Palo Alto, CA, USA) equipped with a fibre optic probe [30]. Emission spectra were recorded with excitation 450 and 530 nm (for *M. cavernosa*) or 435 and 530 nm (*Echinophyllia* sp.). Excitation spectra were collected using the 620 nm emission band. Six defined areas on each colony were used as replicate measurements. Fluorescence micrographs were obtained with a camera-equipped wide-field fluorescence microscope (MZ10 F, Leica Microsystems, Wetzlar, Germany) using a GFP Plus filter.

### 4.3. Protein Expression and Purification

Competent *Escherichia coli* cells were transformed with plasmids (pQE30, pGEM-t) containing the coding sequences for the functional expression of the pcRFPs McavRFP, EosFP, and EechRFP, and grown on LB-ampicillin agar plates. The 5 most fluorescent colonies of each protein type were used to inoculate 300 mL 2xYT liquid cultures; the cultures were incubated overnight at 30 °C, then transferred to 4 °C and shaken at 135 rpm in the dark for 11 to 17 days until they exhibited strong green fluorescence [9]. Harvested cells were disrupted by sonication and centrifuged at 7000 rpm at 4 °C for 10 min. Proteins were purified from the supernatant by Immobilised Metal Ion Affinity Chromatography (IMAC) using TALON^®^ Metal Affinity resin (Clontech, Mountain View, CA, USA); the eluate was desalted in Amicon centrifugal filters (Merck Millipore, Darmstadt, Germany) and re-buffered in TRIS (50 mM, 300 mM NaCl), pH 8.2 to recreate the internal pH of live coral colonies [53,54]. The solutions were diluted in the same buffer until optical density values at 505 nm reached 0.12 to minimise inner filter effects from self-shading of the chromophores in the solution.

### 4.4. Photoconversion and Spectroscopy of Purified Proteins

Field measurements of spectral irradiance in the Red Sea were obtained as courtesy of Eyal et al. [33]. Irradiance values at each measurement wavelength were converted into photon flux following Equation (1):

(1) $$\Phi_{P}=\frac{E_{e}}{E_{P}\times N_{A}}$$

where Φ*_P_* is the photon flux in μmol·m^−2^·s^−1^, *E_e_* is the irradiance at measurement wavelength in W·m^−2^, *E_P_* is the energy of a photon at measurement wavelength (calculated following Planck-Einstein relation) in J, and *N_A_* is Avogadro’s number. For each depth level in the dataset, the spectrum of the 412 nm LED was scaled to match the natural values at peak wavelength to determine the LED photon flux required to simulate the light environment at the respective depth. LED total photon fluxes were then inferred by integrating this curve.

Aliquots (375 μL) of the purified protein solution were placed in a 500 μL quartz cuvette in a fluorescence spectrophotometer (Cary Eclipse, Varian, Palo Alto, CA, USA). The 412 nm LED strip was placed directly above the cuvette holder and photon flux was adjusted with a controller (TMC, London, UK) to match the corresponding value in the natural depth profile. The aliquots were then irradiated for 120 min and red fluorescence emission spectra were recorded at 1 min intervals using 530 nm light provided by the spectrometer for excitation. The conversion experiments were repeated 3 times per protein for each simulated depth level. Maximal fluorescence emission values at 580 nm (EosFP) or 581 nm (McavRFP and EechRFP) were plotted against time. The increase in fluorescence over time could be best-fitted with linear models and the resulting slopes were used to infer photoconversion rates and to fit a linear regression after natural logarithm transformation.

For comparison of photoconversion efficiency of the 412, 448 and 476 nm spectral bands, the respective LED strip was in turn placed above the cuvette and intensity was set to 60 μmol photons·m^−2^·s^−1^; for the 412 nm LED, this value corresponded to the amount of light in the same spectral band that would be received by corals at ~7 m depth. Three replicate conversion experiments per protein type and spectral band were performed. Red emission spectra (excitation 530 nm) were recorded every 15 min for 1 h. During measurements, the LEDs were switched off to avoid additional excitation by the 448 and 476 nm light sources.

### 4.5. Photoconversion Potential of Light Sources

For each depth in the Red Sea field photon flux dataset, the published action spectrum of photoconversion for EosFP [20] was used to calculate the relative probability that a photon in the light field would trigger a photoconversion event. These probabilities were fitted with a log-linear model to obtain an equation relating photoconversion potential with depth. The same probability calculation was applied to the spectra of the 412, 448, and 476 nm LEDs (Figure S1a, 60 μmol photons·m^−2^·s^−1^) and to the spectrum of a metal halide (Figure S1b, 200 μmol photons·m^−2^·s^−1^); these probabilities were then used with the previously obtained equation to calculate an equivalent depth of photoconversion potential for each of our experimental light sources.

### 4.6. Estimation of FRET in Live Colonies

Following the exposure to 476 nm light for 123 days, *M. cavernosa* was placed under a 412 nm LED strip with photon flux set to 60 μmol photons·m^−2^·s^−1^ (12 h:12 h, light:dark) and photoconversion was monitored over 10 days. Emission spectra were collected with excitation wavelength set to 450 and 530 nm. Normalised 514 nm emission (ex = 450 nm) and 582 nm emission (ex = 530 nm) were used as proxy for green and red chromophore concentrations, and fitted with integrated second order rate reaction models using non-linear least squares for parameterisation.

In order to quantify FRET at different stages of photoconversion, we calculated the FRET-derived emission intensity for each set of in vivo measurements following the approach of Müller et al. [50], Equation (2):

(2) $$\mathrm{FRET}=I_{F}-\alpha I_{A}-\beta I_{D}$$

where *I_F_* is the FRET-sensitised donor emission, α*I_A_* is the acceptor spectral bleed through caused by direct excitation of the acceptor by donor excitation wavelength, and β*I_D_* is the donor spectral bleed through caused by direct emission of the donor at the acceptor emission wavelength. For each measurement in the dataset we used 582 nm emission (ex = 450 nm) as *I_F_*, 582 nm emission (ex = 530 nm) as *I_A_*, and 514 nm emission (ex = 450 nm) as *I_D_* (*n* = 5).

In order to estimate donor and acceptor spectral bleed through, we measured *I_F_*, *I_A_*, and *I_D_* for fully unconverted and fully converted samples of purified pcRFPs. These values were then used to calculate the parameters α and β according to Equations (3) and (4) [49,50]:

(3) $$\alpha =\frac{I_{F}}{I_{A}}$$

where *I_F_* and *I_A_* are measured for fully converted protein;

(4) $$\beta =\frac{I_{F}}{I_{D}}$$

where *I_F_* and *I_D_* are measured for fully unconverted protein. The same approach was applied to calculate the FRET-derived emission for *M. cavernosa* exposed to 412 nm LED during the 123 days time series.

### 4.7. Evaluation of Wavelength Conversion In Vitro

In order to compare the potential for wavelength conversion of pcRFPs with that of other cnidarian RFPs, we performed spectroscopic measurements on aliquots of purified EosFP (Stage 1: unconverted; Stages 2–4: partially converted) and eqFP611 [9]. Partial photoconversion was achieved by placing aliquots under 412 nm LED and monitoring absorbance every 5 min, until the desired red:green ratios were achieved. Both proteins were diluted in PBS pH 7.4 until absorbance at 506 nm reached a value of 0.1; absorbance spectra of 100 μL aliquots were measured with a spectrophotometer (Cary 50 Scan, Varian, Palo Alto, CA, USA) in a 10 mm quartz cuvette (3 replicate measurements) and blank-corrected. Fluorescence spectra of the same aliquots were measured in a fluorescence spectrophotometer (Cary Eclipse, Varian, Palo Alto, CA, USA) with excitation wavelength set to 506 nm (3 replicate measurements) and blank-corrected. Two integrated fluorescence values were calculated, one in the yellow to red range ($\int_{550}^{700}f(\lambda )d\lambda$) and one in the yellow-amber range ($\int_{560}^{610}f(\lambda )d\lambda$). Integrated fluorescence values were normalised to μg functional protein, calculated from the measured absorbance after spectral decomposition and the published extinction coefficients of each chromophore [9,20].

## Supplementary Materials

Supplementary materials can be found at www.mdpi.com/1422-0067/18/7/1174/s1.
