# Supplementary material for: FRET-Mediated Long-Range Wavelength Transformation by Photoconvertible Fluorescent Proteins as an Efficient Mechanism to Generate Orange-Red Light in Symbiotic Deep Water Corals
Source: Int J Mol Sci. 2017 Jul 4;18(7):1174. doi: 10.3390/ijms18071174 (PMC5535822; doi:10.3390/ijms18071174)
Supplement: Supplementary file 1 [file ijms-18-01174-s001.zip › ijms-192354-supplementary.pdf]

SUPPLEMENTARY INFORMATION

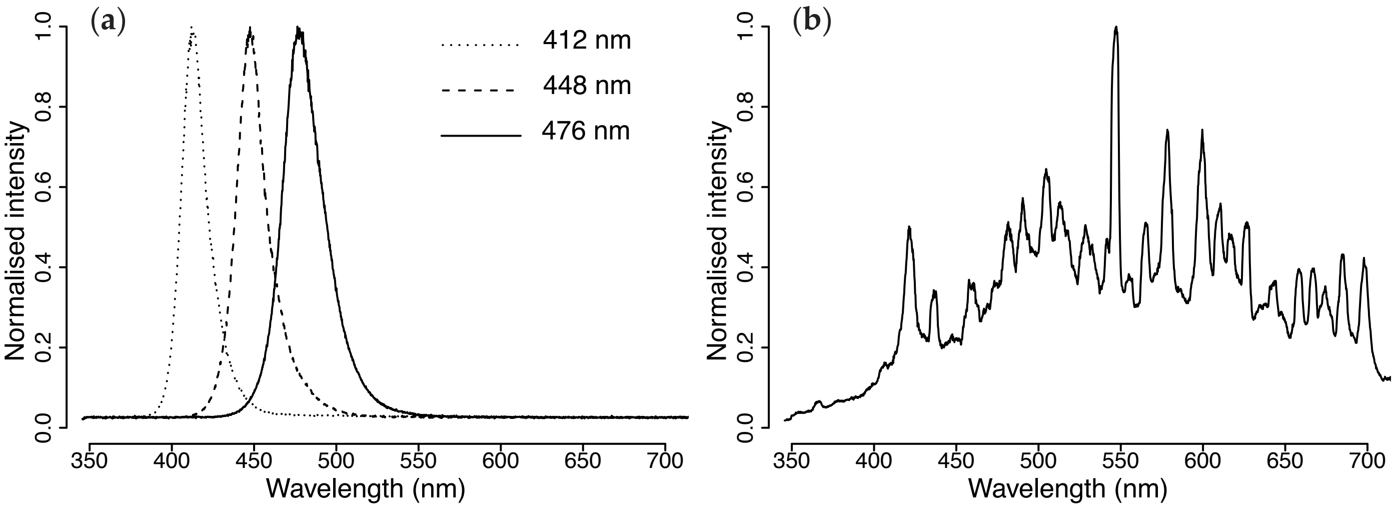

Figure S1. Spectra of light sources used for all experiments. (**a**) Spectra of 412 nm (Aquaray NUV), 448 nm (Aquaray Fiji Blue) and 476 nm LED (Aquaray Reef Blue). (**b**) Spectrum of 400 W metal halide lamp.

Table S1. (**a**) Measured field photon flux, calculated 412 nm photon flux and linear regression results for each depth; (**b**) details of regression analysis for slopes as a function of depth.

(**a**)

			McavRFP			EosFP			EechRFP		
Depth (m)	Tot. ph.f.[1]	412 nm ph.f.[2]	Slope	R^2 adj	p	Slope	R^2 adj	p	Slope	R^2 adj	p
10	525	30.70	4.19	0.99	<0.001	3.01	1.00	<0.001	5.31	0.99	<0.001
20	219	14.60	2.72	0.99	<0.001	1.47	1.00	<0.001	3.16	1.00	<0.001
30	108	7.30	1.32	1.00	<0.001	0.72	0.99	<0.001	1.78	0.99	<0.001
40	74.4	5.29	0.89	1.00	<0.001	0.45	0.98	<0.001	1.13	0.99	<0.001
50	39.5	2.65	0.46	0.99	<0.001	0.17	0.99	<0.001	0.49	1.00	<0.001
60	20.4	1.30	0.22	0.96	<0.001	0.13	0.99	<0.001	0.37	1.00	<0.001
80	7.15	0.44	0.10	0.94	<0.001	0.04	0.81	<0.001	0.17	0.99	<0.001

1. Tot. ph.f = total photon flux (μmol photons $m^{-2}s^{-1}$)
2. 412 nm ph.f = 412 nm photon flux (μmol photons $m^{-2}s^{-1}$)

(**b**)

	Model	R^2 adj	p
**McavRFP**	$e^{2.02 - 0.06 \times depth}$	0.99	<0.001
**EosFP**	$e^{1.59 - 0.06 \times depth}$	0.99	<0.001
**EechRFP**	$e^{2.11 - 0.05 \times depth}$	0.99	<0.001

Table S2. Model equations and statistical test results for non-linear least squares analysis of normalised green and red fluorescence of *M. cavernosa* during photoconversion (Figure 5a).

	**Model**	**k Estimate**	**SE**	**t-value**	**p**
**Green Emission**	1/(k*x+1)	0.025	0.003	9.163	<0.001
**Red Emission**	y_0+1-(1/k*x+1)	0.026	0.002	13.06	<0.001

Table S3. Absorbance maxima (Abs) after spectral decomposition of purified FPs used in Figure 6, and calculated concentration (Conc.) values.

	**Green Chromophore**		**Red Chromophore**		
**Protein**	Abs	Conc. μgμL^{-1}	Abs	Conc. μgμL^{-1}	
EosFP Stage1	0.108	0.039	-	-	0.039
EosFP Stage2	0.11	0.039	0.021	0.013	0.052
EosFP Stage3	0.1	0.036	0.05	0.031	0.067
EosFP Stage4	0.067	0.024	0.149	0.094	0.118
eqFP611	-	-	0.472	0.158	0.158
